# Commentary: Association between methylmalonic acid and cognition—A systematic review and meta-analysis

**DOI:** 10.3389/fped.2022.1048969

**Published:** 2022-11-25

**Authors:** Mahesh Kumar Balasundaram, Dhyuti Gupta, Alok Singh

**Affiliations:** ^1^ Department of Pharmacology, All India Institute of Medical Sciences Raipur, Raipur, Chhattisgarh, India; ^2^ Department of Pharmacology, Teerthanker Mahaveer Medical College and Research Centre, TMU, Moradabad, Uttar Pradesh, India

**Keywords:** pediatric neurology, vitamin B deficiency 12, cognition, systematic reviews, methylmalonic acid

Dear Editor,

We have meticulously read the article “Association Between Methylmalonic Acid and Cognition: A Systematic Review and Meta-Analysis” by Wang et al., published in your esteemed journal (1). The objective of the study was fascinating as the authors tried to explore the correlation between the elevated levels of methylmalonic acid (MMA) and decline in cognition, which is an untouched area in research.

Upon reading the article, there is a slight ambiguity about certain aspects that requires clarification. It is appreciable that the authors addressed the paucity of studies on the aforementioned research question, by including cross-sectional as well as case-control studies apart from randomized controlled trials in this meta-analysis. However, the PICOS criteria for performing the research remains unsatisfactory for the readers, as the study encompassed a wide range of populations (general population and patients) and included extremes of age (infants and the elderly) as well. The scale used to assess cognition was also not uniform among the six cross-sectional studies included in the study. The authors combined the scales that yield continuous data (Mini-Mental Status Examination, Digit Symbol Coding [DSC] Test, Stroop neuropsychological screening tools) with a scale that yields dichotomous data (Bayley—III), which does not seem to be an appropriate step in the methodology (Table 1). Another concern is that the authors failed to demarcate the high and low values of MMA in the methodology section.

**Table 1:** Details of different scales used in study.

Scale	Score range	Application	Interpretation
MMSE	0–30	• Assess cognitive impairment in elderly • Screening for dementia	• Severe cognitive impairment: 0–17 • Mild cognitive impairment: 18–23 • No cognitive impairment: 24–30
Digit symbol coding test		• Assess cognitive function (the comprehension speed)	• This task consists of rows containing small blank squares, each paired with a randomly assigned number from one to nine • Above these rows is a printed key that pairs each number with a different symbol • Using the reference key, the examinee has 120 s to pair specific numbers with given geometric figures.
Stroop neuropsychological screening tools		• To assess the individual's ability to selectively process only one visual feature at a time while inhibiting the processing of other features, which makes it a test of “concentration effectiveness”	• Two forms – Form C and Form C-W • The Form C stimulus sheet consists of 112 colour names (i.e., red, green, blue, and tan) arranged in four columns of 28 names • 120 s are allowed for each stimulus sheet for a maximum test time of 4 min • A score of 99 was determined to have the highest hit rate for discriminating brain-damaged individuals from the normative sample for the 18–49 age group • For the 50+ group a score of 62 produced the highest hit rate
Bayley—III		• Assessment tool for diagnosing developmental delays in early childhood	• Scoring is Dichotomous (1, 0) in BSID III • Scoring is software based in BSID III • Five domains: ○ Cognitive scale – 91 items ○ Language scale – 49 items in the receptive and 48 items in the expressive domain ○ Motor scale – 66 items in the fine motor and 72 items in the gross motor domain ○ Social–Emotional Scale derived from Greenspan Chart ○ Adaptive behavior scale derived from ABAS (adaptive behavior assessment system)

In the study by Kobe et al., two groups were created and compared on the basis of serum vitamin B_12_ levels and not MMA (2). During the data synthesis, the authors probably duplicated that same data. Similarly, in the study by Lildballe et al., the values of MMA have been expressed as median and range. However, the authors, in their meta-analysis, have expressed the values as mean and standard deviation without explaining the method of converting median and range to mean and standard deviation (3). In the study by Bailey et al., the results of the DSC test were presented as least-square mean and standard error, and the authors have not explained the method used to convert the data to mean and standard deviation (4). The authors included the cross-sectional studies to evaluate the influence of MMA as a single factor on cognition, and draw a more objective conclusion; however, with the help of the cross-sectional studies, it is difficult to establish a causal relationship as only a single measurement is performed (5). Among the meta-analysis of randomized studies, combining infants and the geriatric population with two different scales is not a prudent idea and will give erroneous estimates.

The results would have been much more relevant had the meta-analysis been performed considering a uniform population and comparable endpoints. Nevertheless, the authors have instigated the minds of keen researchers to further explore the current topic.
